# Supplementary material for: Impact of COVID-19 on Sedation Requirements during Veno-Venous Extracorporeal Membrane Oxygenation for Acute Respiratory Distress Syndrome
Source: J Clin Med. 2023 May 17;12(10):3515. doi: 10.3390/jcm12103515 (PMC10219298; doi:10.3390/jcm12103515)
Supplement: Supplementary file 1 [file jcm-12-03515-s001.zip › jcm-2381974-supplementary.pdf]

Supplementary Materials

Table S1. – Quartiles of single substances

Parameters	***Sufentanil*** *µg/kg/h*	***Propofol*** *mg/kg/h*	***Midazolam*** *µg/kg/h*	***Isoflurane*** *ml/kg/h*	***Esketamine*** *mg/kg/h*	***Clonidine*** *µg/kg/h*	***Dexmedetomidine*** *µg/kg/h*
25th percentile (“low”; 1 point*)	0.35	0.57	0.40	0.02	0.26	0.50	0.30
50th percentile (“moderate”; 2 points*)	0.50	1.32	27.52	0.03	0.49	0.69	0.52
75th percentile (“high”; 3 points*)	0.66	2.13	54.00	0.05	0.74	0.95	0.70
100th percentile (“very high”; 4 points*)	1.72	6.08	333.27	0.13	2.31	2.62	1.37

* Points referred to the sedation sum score
